# The complete chloroplast genome of *Gaillardia pulchella* Foug. and its phylogenetic analysis

**DOI:** 10.1080/23802359.2025.2550611

**Published:** 2025-08-23

**Authors:** Hongqin Li, Jicheng Yuan, Guiyuan Wu, Liqiang Wang

**Affiliations:** a College of Pharmacy, Heze University, Heze, Shandong Province, P. R. China; b Faculty of Chinese Medicine, Heze Medical College, Heze, Shandong Province, P. R. China; c Production Department, Shandong Buchang Pharmaceuticals Co., Ltd, Heze, Shandong Province, P. R. China

**Keywords:** *Gaillardia pulchella*, Helenieae, chloroplast genome, phylogenetic analysis

## Abstract

*Gaillardia pulchella*, an annual Asteraceae herb native to tropical America, is widely cultivated in China for its vibrant blooms and long flowering period. The species exhibited notable biological activities, including antioxidant and anti-inflammatory effects. We presented the first complete chloroplast genome of *G. pulchella*, spanning 151,778 bp with an overall GC content of 37.7%, and containing 128 genes (85 protein-coding genes, 35 tRNA genes, and eight rRNA genes). Phylogenetic analysis revealed its close relationship with *Galinsoga parviflora*, *Tagetes erecta*, and *Flaveria bidentis*. This study provided valuable genomic insights for evolutionary studies, and potential pharmaceutical applications of *G. pulchella*.

## Introduction

*Gaillardia pulchella* is an annual herbaceous plant belonging to the Asteraceae family, which is native to tropical America and is widely cultivated in China as an ornamental species. In addition to their horticultural value, *G. pulchella* has been reported to contain bioactive compounds, including sesquiterpene derivatives (Kupchan et al. 1966; Yu et al. 1988) and phenolic compounds such as flavones (Moharram et al. 2017). These compounds exhibit various pharmacological properties, including antioxidant, anti-inflammatory, and hepatoprotective effects (Inayama et al. 1984; Harimaya and Inayama 1990).

Despite its medicinal potential, comprehensive genomic investigations of *G. pulchella* remain scarce. Although several nuclear and chloroplast genome markers (e.g. *ndh*F and *trn*L-*trn*F) are accessible in GenBank, a complete chloroplast genome has not been documented to date. Chloroplast genomes are instrumental in phylogenetic reconstruction, species identification, and evolutionary analysis owing to their conserved structure and maternal mode of inheritance. Accordingly, the present study aims to sequence and annotate the complete chloroplast genome of *G. pulchella*, thereby supporting future research on its genetic diversity, evolutionary affiliations, and prospective applications in medicine and agriculture.

## Materials and methods

**Figure 1.**
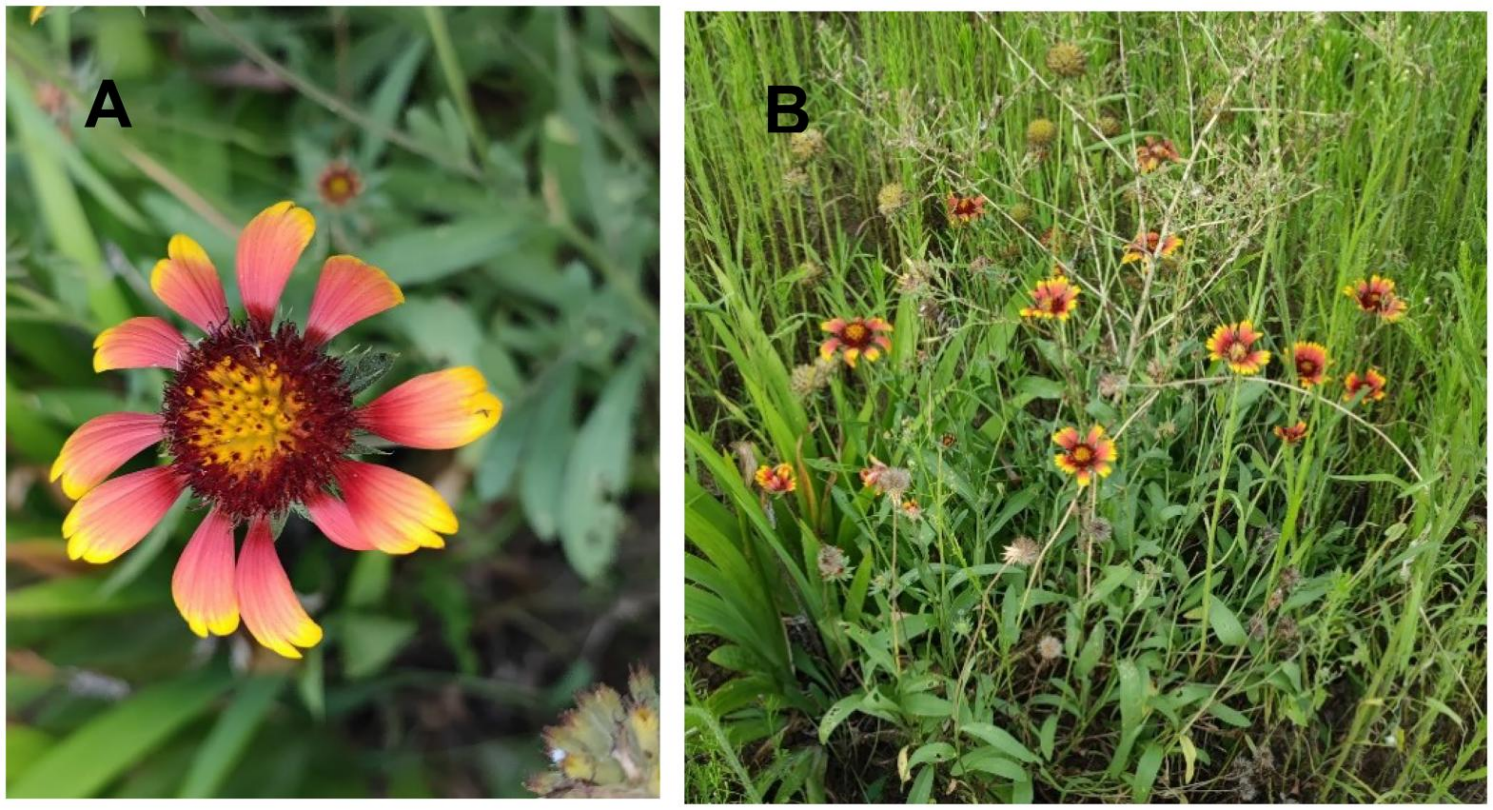
Field photograph of *Gaillardia pulchella*. Location: 35°18’55.37"N, 115°30’38.77"E. Photographer: Liqiang Wang. Key morphological features: Entire plant covered with soft hairs. Leaves alternate, variable in shape (oblong, lanceolate, or spatulate); leaf margins entire or pinnately lobed at base; terminal capitulum with long peduncles; ligulate flowers yellow with brownish-purple bases; flower apices with 2–3 teeth; achenes hairy at base. Panel A: whole plant panorama, panel B: top-down view of the capitulum.

The fresh leaves used for sequencing were obtained from Peony District, Heze City, Shandong Province, China (35°18’55.37′’N, 115°30’38.77′’E) (Figure 1), and a sample was deposited in the Heze University Herbarium (contact person: Hongqin Li, 463056627@qq.com) under specimen number HZ20220809.

Whole genomic DNA was extracted using the Plant Genomic DNA Kit (Tiangen Biotech, Beijing, China). The extracted DNA was fragmented into about 300 bp short-insert fragments to construct sequencing libraries, which were subsequently sequenced on the Illumina NovaSeq 6000 platform at Wuhan Benagen Technology Company Limited, Wuhan, China. Trimmomatic (v0.35) (Bolger et al. 2014) with default settings was employed to filter raw reads by removing adapters and low-quality bases. Following quality control, about 24 GB of clean reads were assembled using GetOrganelle (v1.7.1) (Jin et al. 2020). The complete chloroplast genome was annotated using the online tool CPGAVAS2 (Shi et al. 2019) and manually refined using Apollo (Pontius 2018). The annotated chloroplast genome was subsequently submitted to GenBank under the accession number OR124734. The CPGview tool was applied to visualize the circular genome map of the newly assembled chloroplast genome (Liu et al. 2023).

To investigate the phylogenetic position of *G. pulchella*, the chloroplast genomes of 18 species of the Asteraceae were downloaded from the GenBank database. In addition, the outgroup was *Nymphaea tetragona*. The entire chloroplast genome sequence was aligned using MAFFT software with default parameters (https://mafft.cbrc.jp/alignment/software/) (Katoh and Standley 2016). A maximum-likelihood (ML) phylogenetic tree was subsequently constructed using IQ-TREE (v2.0) (Nguyen et al. 2015) with the best-fit model of TVM+G4, along with 1000 bootstrap replicates.

## Results

**Figure 2.**
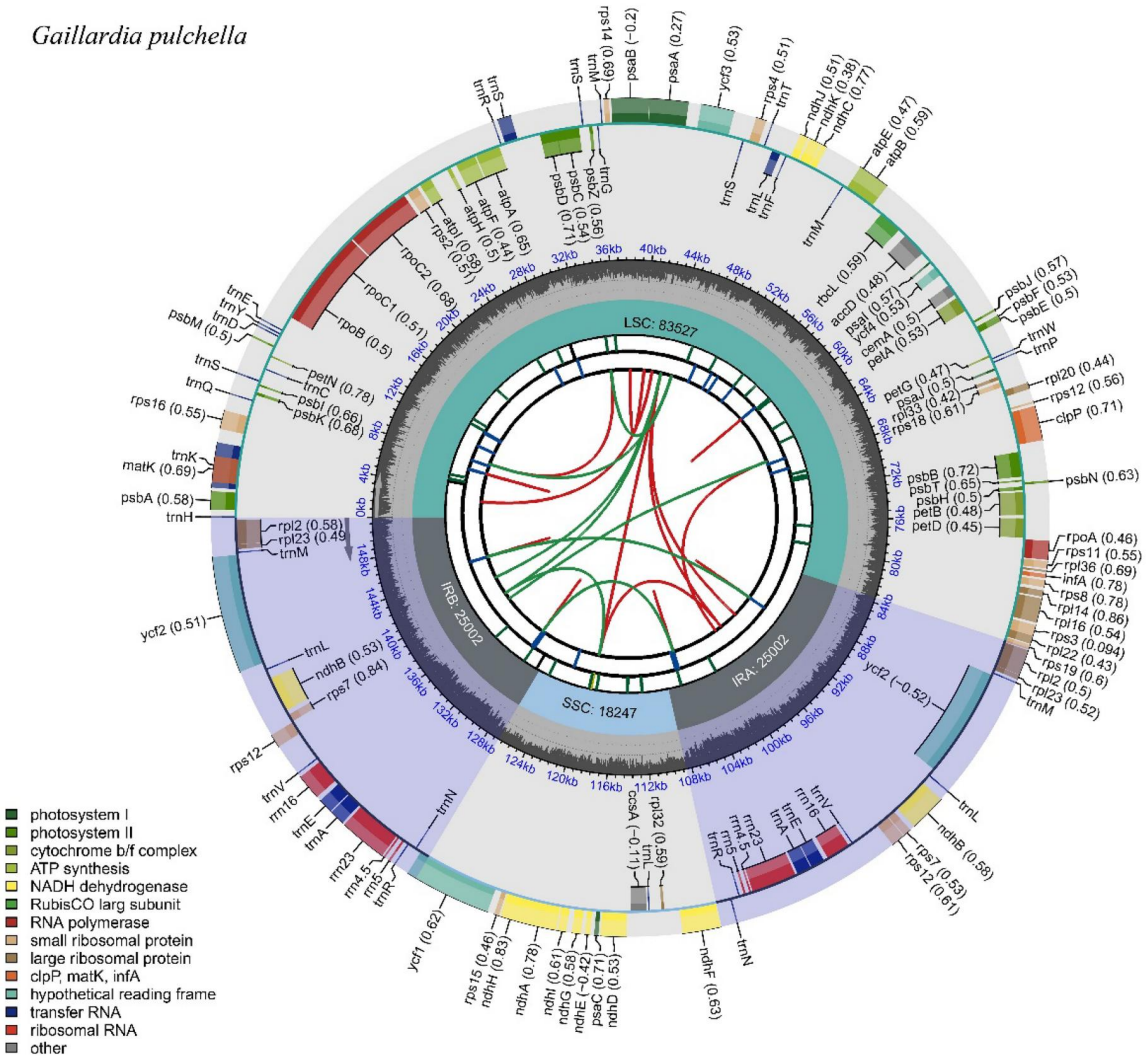
The complete chloroplast genome of *Gaillardia pulchella*. The map contains six tracks. From the center outward, the first track displays the dispersed repeats. The second track shows long tandem repeats as short blue bars. The third track shows short tandem repeats or microsatellite sequences as short bars with different colors. The small single-copy (SSC), inverted repeat (IRA and IRB), and large single-copy (LSC) regions are shown on the fourth track. The GC content along the genome is plotted on the fifth track. The genes are shown on the sixth track. Genes belonging to different functional groups are color-coded. Genes on the inside and outside of the map are transcribed in clockwise and counterclockwise directions, respectively.

The *G. pulchella* chloroplast genome sequence spans 151,778 bp and displays a typical quadripartite structure. Compared to the chloroplast genome of *Arabidopsis thaliana*, a structural rearrangement has occurred in this species’chloroplast genome (Figure S1). The genome comprises two IR regions of 25,002 bp each, separated by a large LSC region of 83,527 bp and a small SSC region of 18,247 bp (Figure 2). The chloroplast genome exhibits variable GC content, with an overall GC content of 37.7%. The assembled complete chloroplast genome shows an average sequencing depth of 915.55×, with a minimum of 182× and a maximum of 1375× (Figure S2). The IR regions have the highest GC content at 43.1%, followed by the LSC and SSC regions at 35.8% and 31.2%, respectively. The *G. pulchella* chloroplast genome is predicted to encode 128 genes, including 85 protein-coding genes (PCGs), eight rRNA genes, and 35 tRNA genes. Within the IR regions, six unique PCGs (*rps*12, *rps*7, *rpl*2, *rpl*23, *ndh*B, and *ycf*2), seven unique *trn*A genes (*trn*M, *trn*L, *trn*V, *trn*E, *trn*A, *trn*R, and *trn*N), and four unique rRNA genes (*rrn*16S, *rrn*23S, *rrn*4.5S, *rrn*5S) were identified. Across the chloroplast genome, ten PCGs (*rps*16, *rpo*C1, *atp*F, *pet*B, *pet*D, *rpo*A, *rpl*16, *rpl*2, *ndh*B, and *ndh*A) each contain one intron, while two PCGs (*ycf*3, *clp*P) contain two introns. In addition, five tRNA genes (*trn*H-GUG, *trn*K-UUU, *trn*Q-UUG, *trn*S-GCU, and *trn*C-GCA, *trn*D-GUC, and *trn*Y-GUA) contain one intron. The *rps*12 gene was subjected to trans-splicing. The structures of the cis-splicing and trans-splicing PCG genes are shown in Figure S3 and Figure S4, respectively. The lengths of the protein-coding genes, tRNA genes, and rRNA genes in the chloroplast genome are 2,8456 bp, 3,831 bp, and 9,273 bp, accounting for 18.7%, 2.5%, and 6.1% of the total genome length, respectively.

**Figure 3.**
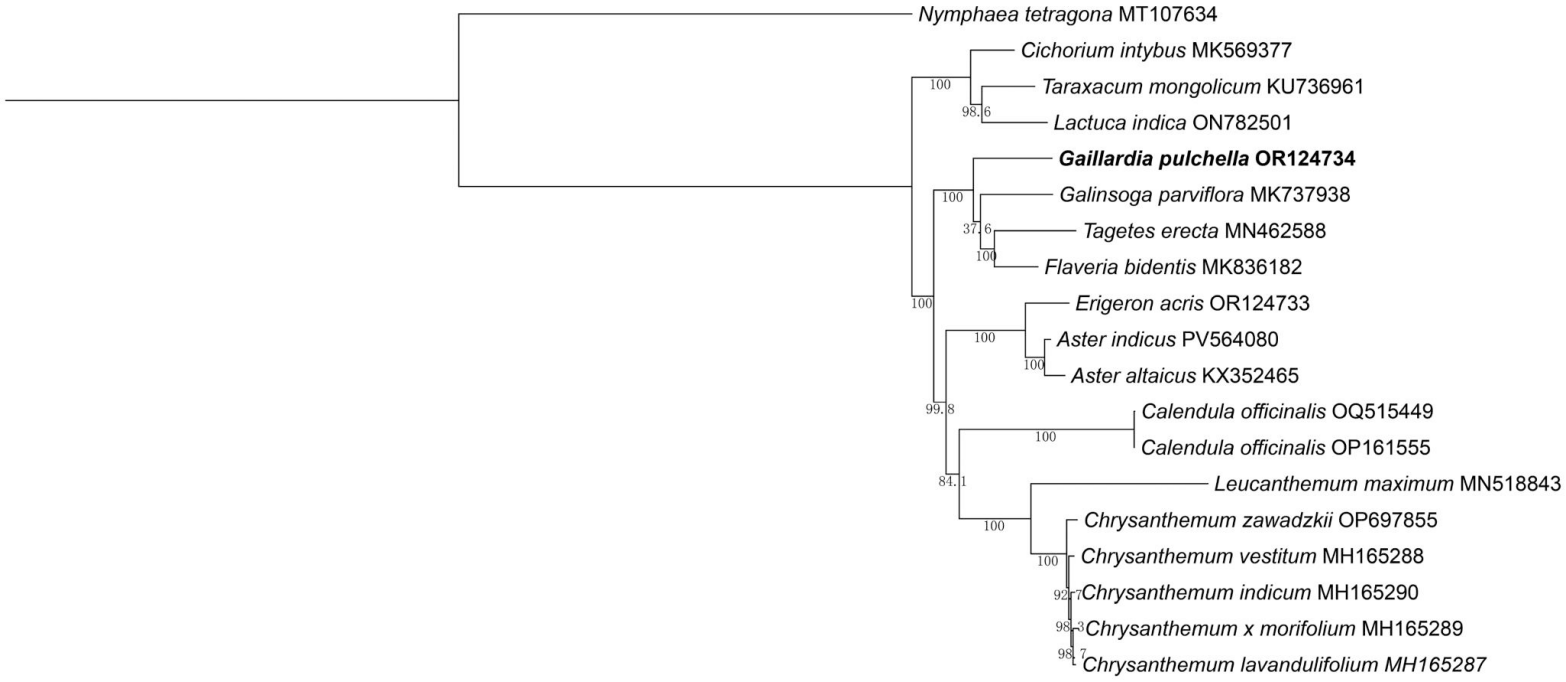
Maximum-likelihood phylogeny of *Gaillardia pulchella* and its close relatives using whole-genome sequences. The bootstrap values based on 1000 replicates are shown on each node in the phylogenetic tree. The 19 species were downloaded from GenBank. *Taraxacum mongolicum* KU736961 (Kim et al. 2016), *Chrysanthemum indicum* MH165290 (Ma et al. 2020), *Chrysanthemum lavandulifolium* MH165287 (Ma et al. 2020), *Chrysanthemum vestitum* MH165288 (Ma et al. 2020), *Flaveria bidentis* MK836182 (Yang et al. 2019), *Leucanthemum maximum* MN518843 (Chen et al. 2019), *Tagetes erecta* MN462588 (Song et al. 2019), *Galinsoga parviflora* MK737938 (Zhang et al. 2019), *Gaillardia pulchella* OR124734 (this study), *Calendula officinalis* OP161555 (Zhang et al. 2024), Calendula officinalis OQ515449, *Nymphaea tetragona* MT107634 (outgroup) (Sun et al. 2021). *Aster indicus* PV564080, *Lactuca indica* ON782501, *Erigeron acris* OR124733, *Aster altaicus* KX352465 (Park et al. 2017), *Chrysanthemum zawadzkii* OP697855, *Cichorium intybus* MK569377 (Yang et al. 2019).

Phylogenetic analysis revealed that *G. pulchella* clustered into a monophyletic group with *Galinsoga parviflora*, *Tagetes erecta*, and *Flaveria bidentis* (Figure 3). This clade received maximum bootstrap support (100%), confirming its close evolutionary relationship.

## Conclusions and discussion

This study provides the first detailed characterization of the genome of *G. pulchella*, revealing a typical annular tetrad structure measuring 151,778 bp and comprising 128 predicted genes. A structural rearrangement event was identified within the chloroplast genome. Phylogenetic analysis demonstrated that *G. pulchella*, *Galinsoga parviflora*, *Tagetes erecta*, and *Flaveria bidentis* form a robustly supported monophyletic clade within the Asteraceae. This clustering is consistent with recent molecular phylogenetic investigations based on chloroplast DNA markers (e.g. *ndh*F and *trn*L-*trn*F) and nuclear ribosomal ITS sequences, which consistently place these genera within the Heliantheae alliance—a lineage defined by radiate capitula and conserved mechanisms regulating floral symmetry (Chen et al. 2018). The chloroplast genome analysis of *G. pulchella* further reinforces its taxonomic placement within the Asteraceae. Accordingly, the phylogenetic results offer a reliable depiction of the evolutionary position of *G. pulchella*.

## Supplementary Material

Supplemental Material

## Data Availability

The complete chloroplast genome sequence of *G. pulchella* in this study has been submitted to the NCBI database under the accession number OR124734 https://www.ncbi.nlm.nih.gov. The associated BioProject, BioSample, and SRA numbers are PRJNA928567, SAMN48024363, and SRR33185918.
