# Supplementary material for: The complete chloroplast genome of Gaillardia pulchella Foug. and its phylogenetic analysis
Source: Mitochondrial DNA B Resour. 2025 Aug 23;10(9):863–7. doi: 10.1080/23802359.2025.2550611 (PMC12377075; doi:10.1080/23802359.2025.2550611)
Supplement: Supplemental Material [file TMDN_A_2550611_SM1668.docx]

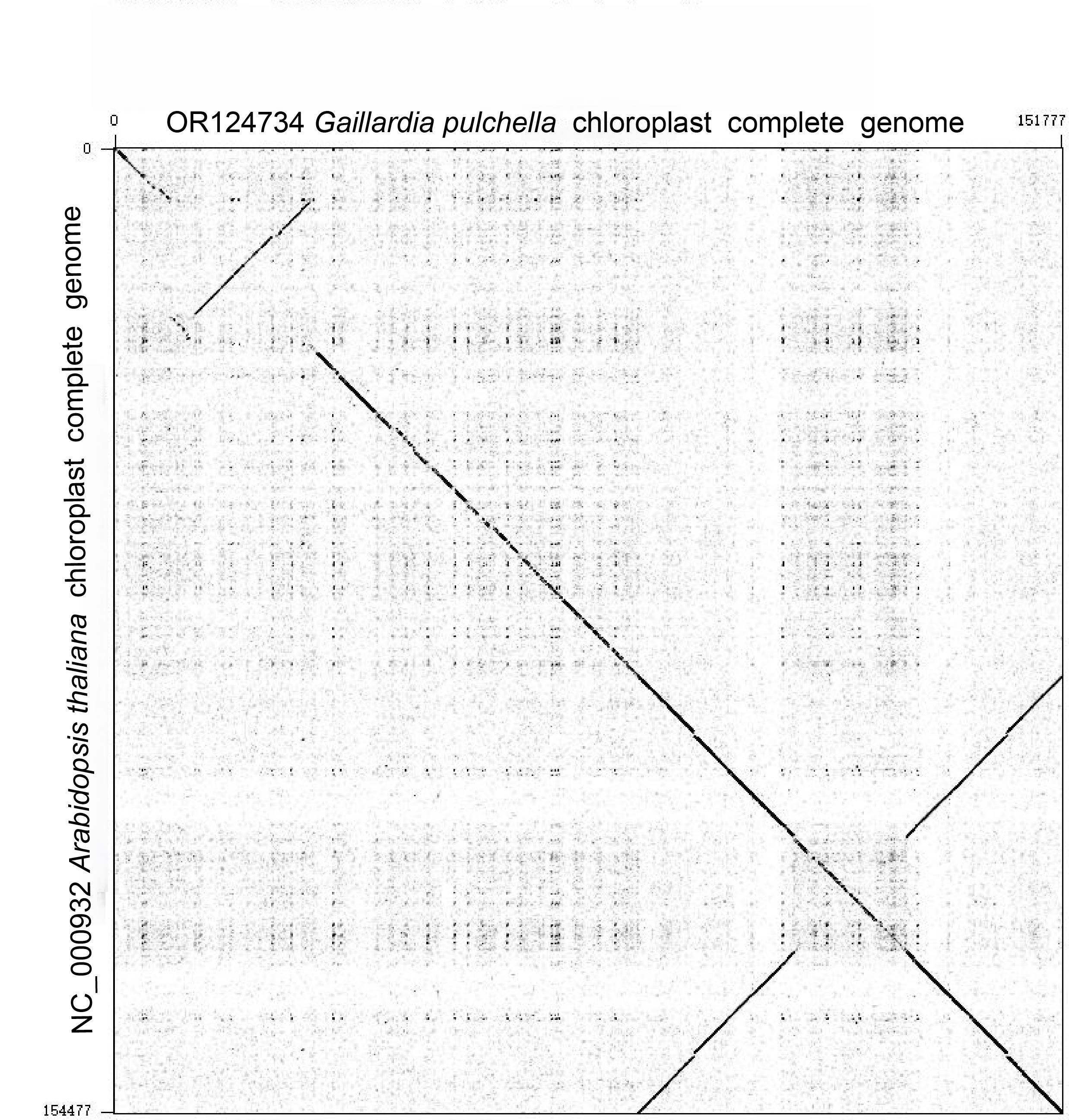


w

**Figure S1. Dotplot map comparing the chloroplast genomes of *Arabidopsis* *thaliana* (NC_000932, Y-axis) and** ***Gaillardia* *pulchella* (OR124734, Y-axis).**

**
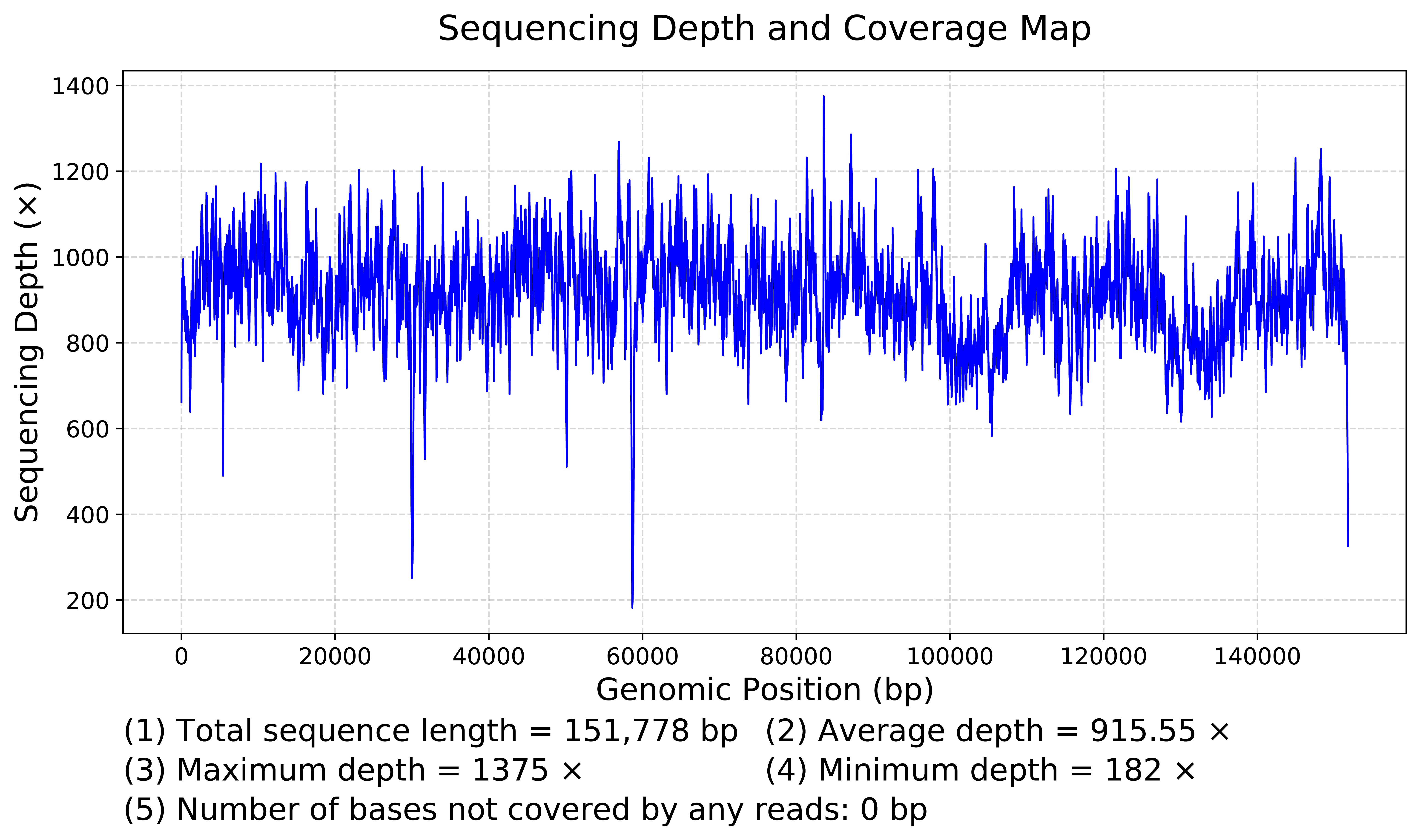
**

**Figure S2 The coverage depth of the chloroplast genome assembly *Gaillardia* *pulchella.***


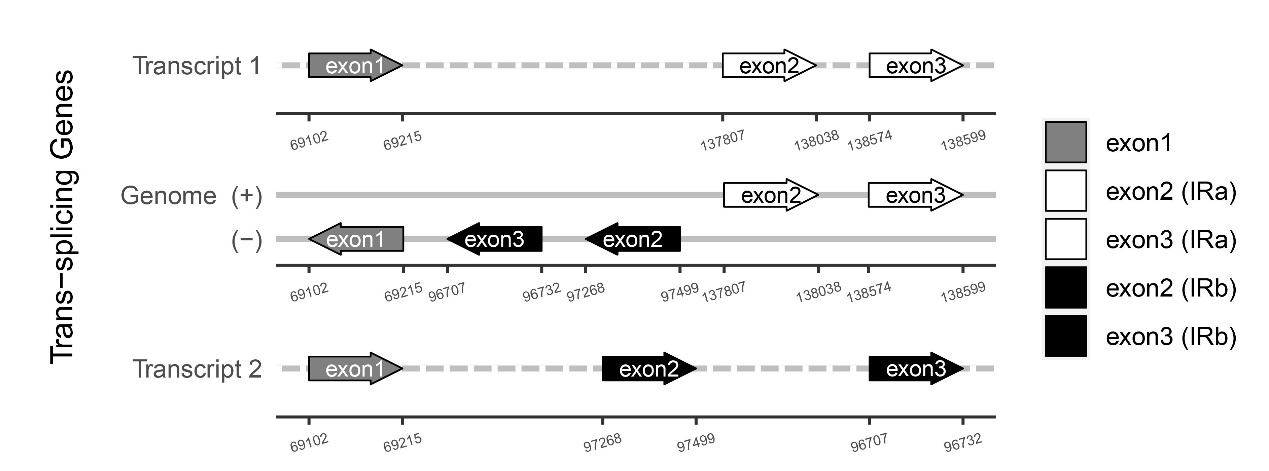


**Figure S3. Schematic map of the trans-splicing gene *rps*12 in the chloroplast genome of *Gaillardia pulchella*.**


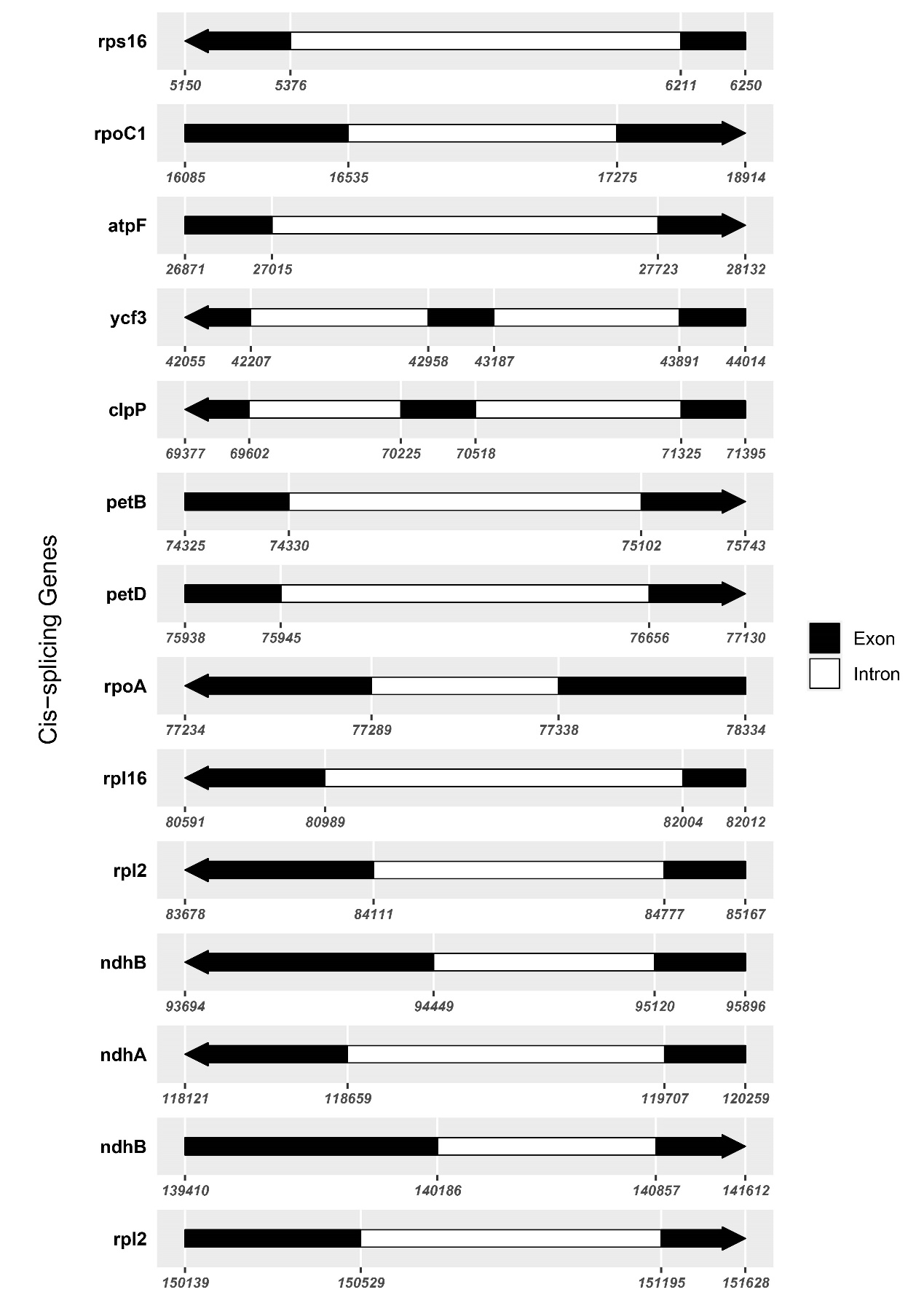


**Figure S4. Schematic map of the cis-splicing genes in the chloroplast genome of *Gaillardia pulchella*.** Exons and introns are shown in black and white, respectively. The arrow indicates the sense direction of the gene.
